# Nitrogen dioxide concentrations in neighborhoods adjacent to a commercial airport: a land use regression modeling study

**DOI:** 10.1186/1476-069X-9-73

**Published:** 2010-11-17

**Authors:** Gary Adamkiewicz, Hsiao-Hsien Hsu, Jose Vallarino, Steven J Melly, John D Spengler, Jonathan I Levy

**Affiliations:** 1 Department of Environmental Health, Harvard School of Public Health, 401 Park Drive, Boston, MA, USA; 2 Department of Environmental Health, Boston University School of Public Health, 715 Albany St., Boston, MA, USA

## Abstract

**Background:**

There is growing concern in communities surrounding airports regarding the contribution of various emission sources (such as aircraft and ground support equipment) to nearby ambient concentrations. We used extensive monitoring of nitrogen dioxide (NO_2_) in neighborhoods surrounding T.F. Green Airport in Warwick, RI, and land-use regression (LUR) modeling techniques to determine the impact of proximity to the airport and local traffic on these concentrations.

**Methods:**

Palmes diffusion tube samplers were deployed along the airport's fence line and within surrounding neighborhoods for one to two weeks. In total, 644 measurements were collected over three sampling campaigns (October 2007, March 2008 and June 2008) and each sampling location was geocoded. GIS-based variables were created as proxies for local traffic and airport activity. A forward stepwise regression methodology was employed to create general linear models (GLMs) of NO_2 _variability near the airport. The effect of local meteorology on associations with GIS-based variables was also explored.

**Results:**

Higher concentrations of NO_2 _were seen near the airport terminal, entrance roads to the terminal, and near major roads, with qualitatively consistent spatial patterns between seasons. In our final multivariate model (R^2 ^= 0.32), the local influences of highways and arterial/collector roads were statistically significant, as were local traffic density and distance to the airport terminal (all p < 0.001). Local meteorology did not significantly affect associations with principal GIS variables, and the regression model structure was robust to various model-building approaches.

**Conclusion:**

Our study has shown that there are clear local variations in NO_2 _in the neighborhoods that surround an urban airport, which are spatially consistent across seasons. LUR modeling demonstrated a strong influence of local traffic, except the smallest roads that predominate in residential areas, as well as proximity to the airport terminal.

## Introduction

People living near large airports may experience elevated exposures to air pollution and ambient noise which can directly affect health and quality of life [1]. Exposure to air pollutants within these neighborhoods may be influenced by: emissions from aircraft activity; emissions from ground support equipment and other sources involved in ground operations, and from traffic in surrounding neighborhoods, partly induced by the airport's presence [2].

Nitrogen dioxide (NO_2_) would be anticipated to demonstrate spatial variability in close proximity to airports, and in general, is of increasing concern in relation to airports and other settings with significant mobile source activity. In the European Union, ambient standards for NO_2 _have recently been tightened, and along with the World Health Organization (WHO), guidelines of a 40 μg/m^3 ^annual average and a 200 μg/m^3 ^1-hour maximum [3] were established. NO_2 _standards in the United States have historically been less stringent (annual average of 100 μg/m^3 ^or 53 ppb), but the National Ambient Air Quality Standards (NAAQS) were recently re-evaluated, with revisions that emphasize the importance of near-roadway concentration gradients and likely imply heightened attention paid to NO_2 _in upcoming years [4].

Spatial patterns of NO_2 _have been characterized in a number of studies using land use regression (LUR), modeling variability in measured concentrations as a function of GIS-based covariates representing traffic and other predictors. These studies have generally involved simultaneous deployment of passive samplers within an urban area, and have explained a majority of spatial variability with covariates including proximity to major roadways or traffic density within buffers surrounding monitors [5-8]. A more limited number of studies have conducted multiple sampling sessions to capture the seasonal variability intrinsic in NO_2 _concentrations, generally using these observations to predict average concentrations across seasons [9-12].

To our knowledge, no LUR studies have been conducted in urban neighborhoods proximate to airports. In these settings, separating the influence of major roadways from activities on the airport grounds may be particularly challenging, given that aircrafts and ground-based mobile sources (i.e., cars, buses and trucks) both emit nitrogen oxides and often co-vary over time. Simultaneously characterizing contributions from roadways and airport activities may require high-density ambient monitoring coupled with detailed meteorological characterization (i.e., wind fields with high spatial and temporal resolution). While atmospheric dispersion models linked with local-scale emissions characterization can also be used to determine relative source contributions, both emissions and dispersion models may have heightened uncertainties at high spatial resolution, and it is valuable to determine insights available through ambient monitoring.

Within this study, we conducted passive sampling of NO_2 _in neighborhoods surrounding T.F. Green Airport in Warwick, Rhode Island, using saturation sampling across multiple seasons with the aim of capturing signals from both local traffic and airport activities in a community proximate to an airport. While other pollutants are clearly contributed by aircraft emissions, we focus on NO_2 _in this investigation to understand the relative contributions of local traffic and airport activities for a pollutant with appreciable spatial variability that can be captured through passive sampling.

## Methods

### Sampling and Analysis

**Figure 1:**
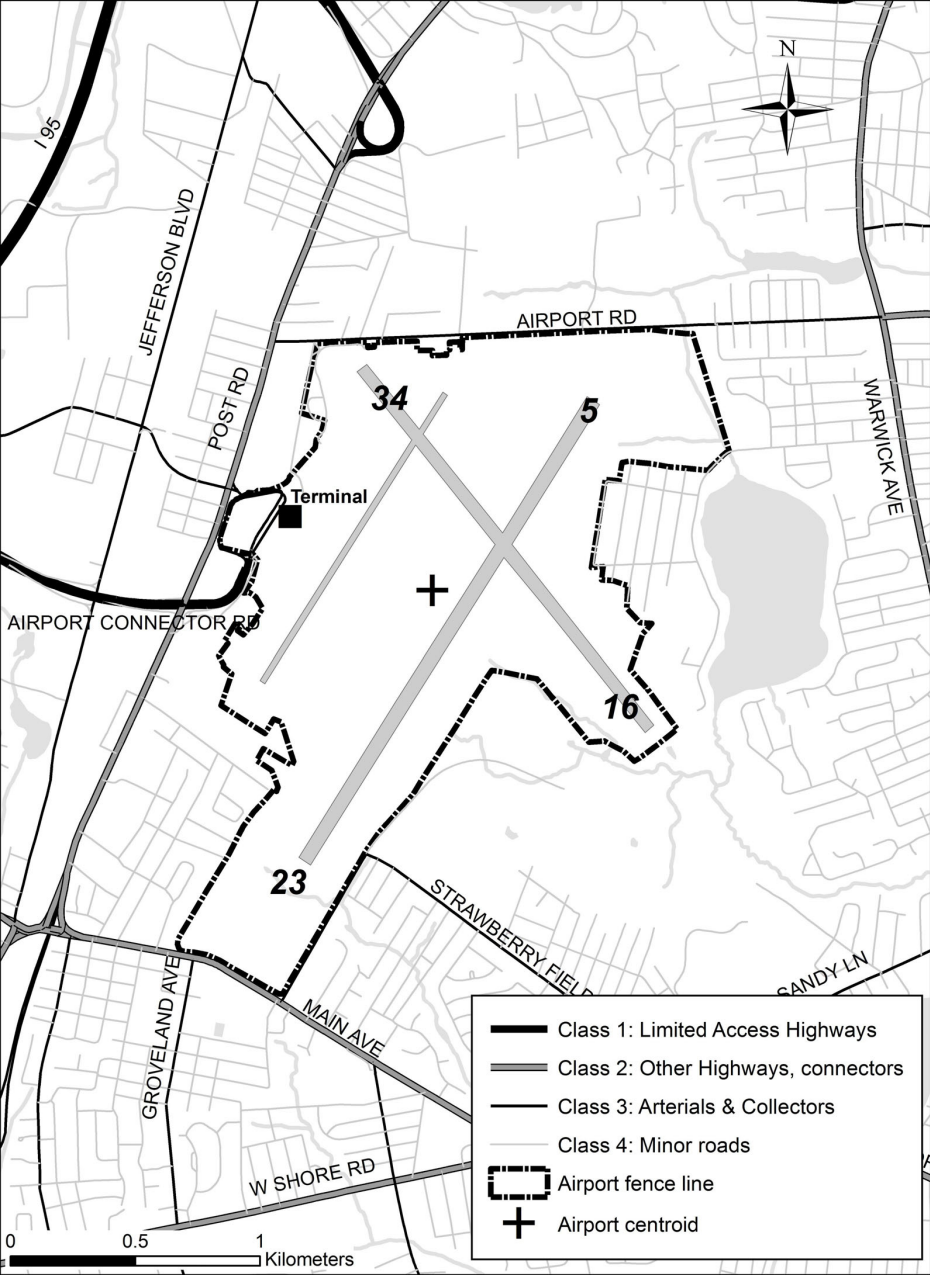
**T.F. Green Airport and surrounding neighborhoods**.

Monitoring was conducted during three sampling campaigns (October 2007, March 2008 and June 2008) at T.F. Green Airport in Warwick, Rhode Island (Figure 1). T.F. Green is a relatively small airport, with approximately 150 arrivals and 150 departures per day, largely occurring on the primary runway (5/23). For the 12-month period ending April 2008, this activity was divided between commercial (45%), air taxi (30%) and general aviation traffic (25%). Road traffic is generally modest on the smaller roads in the neighborhoods surrounding T.F. Green, with significant traffic volume on Interstate 95 (approximately 150,000 vehicles per day), approximately 100,000 vehicles per day on the Airport Connector Road, and between 20,000 and 35,000 vehicles per day on the major roadways surrounding the airport. In general, the major roadways near the airport are largely populated by commercial and retail establishments, and the residential neighborhoods nearby generally consist of single-family homes with relatively low traffic volumes on the surrounding roadways.

Palmes diffusion tube samplers [13] were deployed along the airport's fence line and within the surrounding neighborhoods. Measurements were taken over 14 days in October 2007 (henceforth Session 1) and over 7 days in both March 2008 and June 2008 (Sessions 2 and 3, respectively), given logistical considerations that precluded more rapid sampler pick-up in Session 1. These sampling periods were chosen to examine pollutant patterns over more than one season, to ensure that our findings were robust over time, and coincided with continuous monitoring being conducted in a larger multipollutant study. In total, 695 tubes were deployed over the three sampling campaigns, and latitudes and longitudes (WGS 1984) of each sampling location were recorded using GPS receivers. Both recreational (Etrex Vista, GPSMap60CSx from Garmin) and high accuracy (GeoXT, GeoExplorer3 from Trimble) receivers were used.

Sampling locations were chosen based a few considerations. First, we wished to provide saturation coverage of the fence line surrounding the airport, given our interest in understanding sources within this area, while being limited in our access to active sections of the airport grounds. Any differential concentrations along the fence line that corresponded with winds fetching across the airport grounds would indicate a significant contribution from the airport grounds, and this approach controls for distance from the airport (if not from other sources). In addition, we wished to provide coverage of residential neighborhoods and key local roadways that service the airport and influence local air pollution patterns, with measurements taken in all prevailing wind directions and at a variety of distances from the airport and major roadways. Samplers were placed at heights of 6-8 ft, primarily based on balancing the ease of placement with some concerns about tampering by children in the neighborhood. Within the neighborhoods, samplers were attached to utility poles, and on the fence line, they were physically attached to the chain link fence. Locations along the fence line were approximately evenly-spaced along the accessible portions of the fence.

Of the samplers deployed, 51 were lost or damaged in the field, resulting in 644 sampled locations over the three seasons (253 fence line locations and 391 community or intermediate locations). While sampling density was lower in Session 1, in part because of greater losses due to sampler theft during this session, the spatial extent of sampling was consistent between sessions. Following each sampling session, all Palmes tubes were returned to the Harvard School of Public Health and analyzed with a spectrophotometer using standard methodology [13].

Duplicate and blank samples were used to assess the repeatability and reliability of our NO_2 _measurements. For each sampling session, laboratory blanks (n = 10/session) and field blanks (n = 20-25/session) were analyzed. Low levels of NO_2 _were detected on both types of blanks (mean of 0.01 μg/m^3 ^for laboratory blanks and 0.09 μg/m^3 ^for field blanks). The mean value from laboratory blanks were subtracted from the measured NO_2 _concentrations. Duplicate sampling was conducted on 10-12% of field samples (across sessions), and relative precision was calculated using differences in standard deviation between each pairs of the duplicates, divided by the overall mean concentration. The relative precision of the duplicate NO_2 _measurements ranged from 14.1% to 15% across the three sampling sessions, indicating reasonable precision given the aims of our analyses.

### GIS Variables

ArcMap 9.2 (ESRI, Redlands CA) was used to create GIS-based variables as proxies for local traffic and airport activity. Sampling locations were imported into a personal geodatabase and projected in the Rhode Island State Plane Projection, North American Datum 1983. This same projection was used for all other spatial datasets including 1:5000 roads from Rhode Island Department of Transportation (RI DOT) downloaded from RIGIS http://www.edc.uri.edu/rigis/. The airport fence line and runways were based on ESRI Street Map 9.2 data. The fence line was modified using data collected by GPS in the field. Traffic counts were derived from the 2007 Traffic Flow map from RI DOT and 2001 data from RIGIS http://www.dot.ri.gov/engineering/gis/maps.asp.

As it was unclear *a priori *which GIS-based variables would adequately reflect various source categories, we constructed a number of variables representing proximity to sources and/or source strengths (Table 1). Our primary GIS-based variables reflected: the distances to significant local roadways and convenient airport markers (terminal location, fence line, runways and centroid of airport grounds); kernel-weighted traffic density within 100-400 m of the sampling locations; the length of local roadways by class within 100-400 m of sampling locations; and traffic-weighted roadway lengths within 100-400 m of sampling locations. Local roadways were categorized into four classes, according to a methodology used by RI DOT: Class 1: Limited access highways; Class 2: Other highways and connectors; Class 3: Arterials and collectors; Class 4: Minor roads. For traffic variables, buffers larger than 400 m were considered in preliminary analyses but were discarded given stronger associations with smaller buffers and anticipated spatial gradients of NO_2_.

**Table 1:** GIS variables used in model-building

Variable category/type	Variable(s) (units)	Units
Distance to nearest road class	Distance to nearest Class 1 Roadway (RI DOT)	m
	
	Distance to nearest Class 2 Roadway (RI DOT)	m
	
	Distance to nearest Class 3 Roadway (RI DOT)	m
	
	Distance to nearest Class 4 Roadway (RI DOT)	m

Traffic density	Traffic density within various radii (100, 200, 300, 400 m)	vehicles/day/km^2^

Distance to airport proxies	Distance to airport terminal	m
	
	Distance to Runway 16/34	m
	
	Distance to Runway 5/23	m
	
	Distance to airport centroid	m
	
	Distance to airport fence line	m

Total length of proximate roadways	Total length of Class 1 roadways (within 100-400 m)	m
	
	Total length of Class 2 roadways (within 100-400 m)	m
	
	Total length of Class 3 roadways (within 100-400 m)	m
	
	Total length of Class 4 roadways (within 100-400 m)	m
	
	Total length of all roadways (within 100-400 m)	m

Total length of proximate roadways, weighted by wind rose (fraction of time by direction, 8 segments)	Total length of Class 1 roadways, weighted (within 200 m)	m
	
	Total length of Class 2 roadways, weighted (within 200 m)	m
	
	Total length of Class 3 roadways, weighted (within 200 m)	m
	
	Total length of Class 4 roadways, weighted (within 200 m)	m

Traffic-weighted road length	ADT-weighted road length (within 100-400 m)	vehicle-meters/day

In addition, we were interested in evaluating whether inclusion of meteorological data could provide more informative LUR models, as demonstrated previously for short-term concentrations (though less so for long-term concentrations) [8]. As an initial assessment, we constructed wind-weighted versions of our roadway length variables within 200 m radii. Meteorological measurements were collected using anemometers (RM Young Marine Ultrasonic Anemometers) deployed in close proximity to the airport. While meteorological data were collected at 10-second resolution, we focused in this analysis on meteorology aggregated across the sampling period given our integrated NO_2 _samples. Wind roses were created for each sampling period, dividing wind direction data into eight equal segments, centered on standard compass directions (N, NE, E, SE, S, SW, W, NW). The road length variables were re-estimated based on the length of roadways within each one of these segments and within 200 m of our sampling points. Finally, these road length variables were weighted by the wind rose data, creating a wind-weighted traffic proxy.

### Exploratory Analysis with GIS

In order to explore trends in the data, NO_2 _concentrations for each sampling session were linked to sampling locations and displayed on maps. The ArcGIS 9.2 Geostatistical Analyst Extension kriging function was used to create surfaces displaying NO_2 _concentrations as rasters with a 10 m cell size.

### Modeling Methodology

Our goal was to develop models which describe the association between air pollutant concentrations and GIS-derived spatial parameters and site characteristics; this approach, an alternative to dispersion modeling, has been termed 'land use regression modeling'. Specifically, a modified forward stepwise regression methodology was employed to create general linear models (GLMs) of NO_2 _variability near the airport. A dummy variable for each sampling session was included in all models to account for seasonal variability, noting that the sampling locations were similar but not identical across sessions, making a single model of average concentrations impractical. First, bivariate GLM models that included the 'session' variable were created to identify key explanatory metrics within three broad categories: (a) airport proxies, (b) distances to nearest roads by RI DOT road class, and (c) other proxies for local traffic (Table 1).

Due to the high degree of collinearity in many of the GIS variables and our desire for a parsimonious and interpretable model, our model building was initially guided by exploring relationships between our dependent variable (NO_2 _concentration) and individual variables within the categories listed above. Within each category, we considered only the variable with the strongest association as a candidate for inclusion in larger-order multivariate models. For example, more than one traffic proxy variable constructed from identical underlying data (e.g., road-length weighted traffic density and total traffic density) or those utilizing the same metric at different radii (e.g., 100 m versus 300 m) were not permitted into a single model. Because of our specific interest in airport activity and concerns about collinearity, we first introduced candidate airport proxy variables then tested traffic proxy variables, including distance to road class and proxies for local traffic.

While this structured model-building sequence limits the possibility of spurious associations, our results could be sensitive to this approach, and we conducted a series of sensitivity analyses to test the robustness of our findings. First, we tested substitutions between individual variables within each variable type (e.g., airport proxies, traffic proxies) at each step in the process. We also created models with interaction terms between session and each variable within the final multivariate model, to test possible slope changes by season. As a check on this manual model-building procedure, we also employed an automated forward stepwise regression algorithm in SAS utilizing all significant univariate variables (with p < 0.05 as the inclusion/exclusion criterion). Finally, we considered whether our model would differ if built only using fence line observations or only using non-fence line observations.

## Results

**Figure 2:**
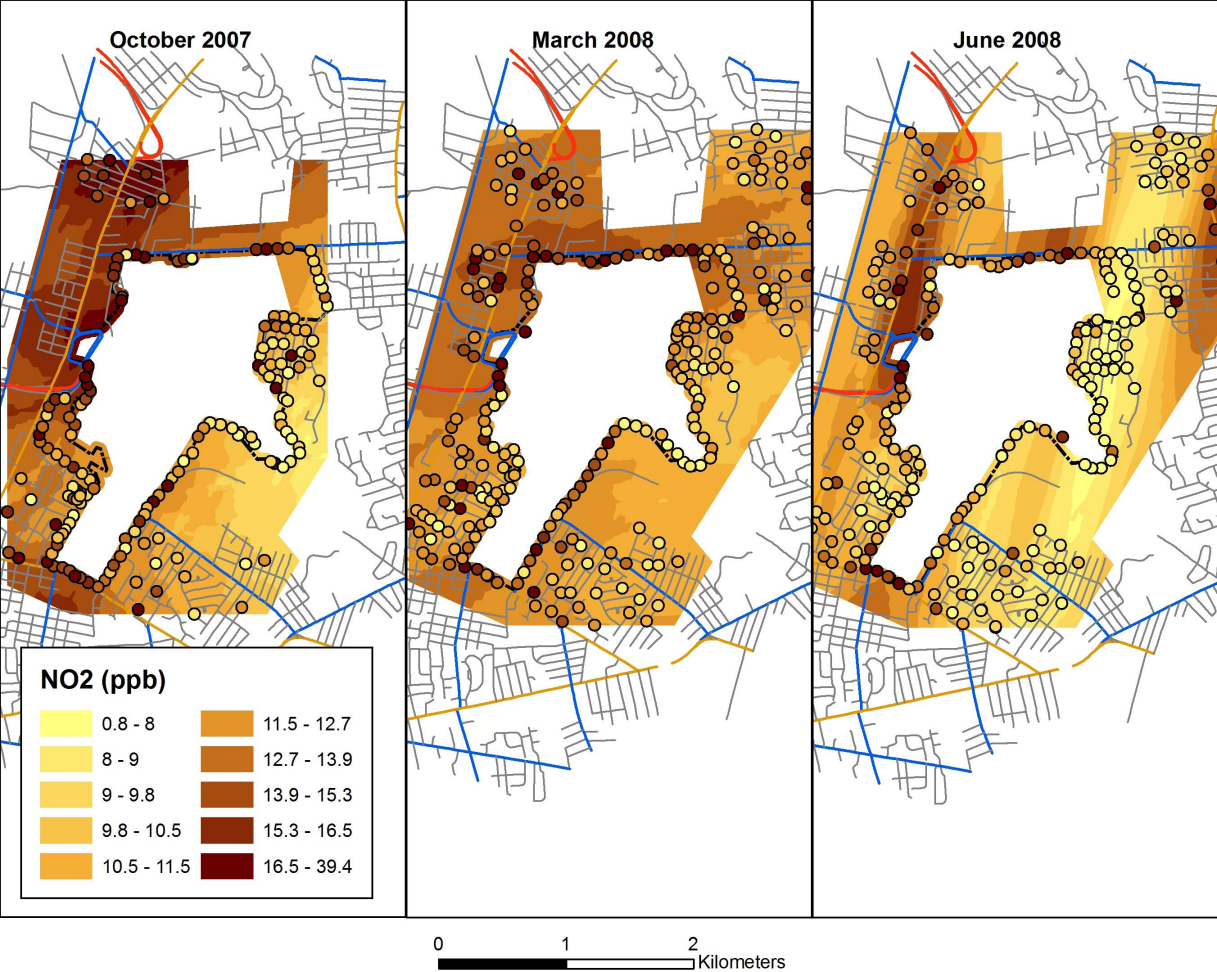
**Measured NO_2 _concentrations during three sampling campaigns, superimposed on smoothed surface created by kriging**. (Cutpoints are quantiles for October 2007 session. The areas within the airport fence line and approximately 200 m away from measured points were masked.)

The mean NO_2 _concentration for all samples was 11.6 ppb (SE = 0.16), with variability across sampling sessions, reflecting seasonal trends (Table 2). These levels are generally consistent with mean NO_2 _concentrations based on data collected at a U.S. EPA monitoring station in Providence, Rhode Island (mean over sampling campaigns = 10.1 ppb). While differential spatial coverage between sessions precludes direct comparisons and spatial surfaces should be interpreted with caution given the visual display of some areas outside of the spatial extent of sampling, general spatial patterns are qualitatively consistent between sessions (Figure 2), with higher concentrations generally detected northwest of the airport and near major roadways. Considering the GIS variables proxying for airport or traffic proximity, values displayed significant variability across our monitoring locations, as anticipated (Table 3).

**Table 2:** NO_2 _Summary Statistics

	Session 1	Session 2	Session 3	All sessions
Date	October 2007	March 2008	June 2008	-

Sampling period	14 days	7 days	7 days	-

N	167	248	229	644

Mean (SE) (ppb)	12.4 (0.3)	12.2 (0.3)	10.4 (0.2)	11.6 (0.16)

Minimum (ppb)	0.8	1.2	3.7	0.8

Median (ppb)	11.6	11.7	9.8	11.0

75^th ^Percentile (ppb)	15.2	13.6	11.8	13.4

95^th ^Percentile (ppb)	18.7	18.4	16.5	17.9

Maximum (ppb)	30.6	39.4	30.0	39.4

**Table 3:** GIS Variable Summary Statistics

Variable/Type	Mean	Median (Range)
**DISTANCE TO NEAREST ROAD CLASS (RI DOT)**		

**Distance to nearest Class 1 Road (m)**	1179.80	1291 (28 - 2221)

**Distance to nearest Class 2 Road (m)**	550.07	431 (9 - 1512)

**Distance to nearest Class 3 Road (m)**	377.70	283 (1 - 1261)

**Distance to nearest Class 4 Road (m)**	354.90	8 (0 - 372)

**TRAFFIC DENSITY WITHIN VARIOUS RADII**		

**Traffic Density within 100 m (vehicle-m/day/km^2^)**	37.39	1.5 (0 - 322.9)

**Traffic Density within 200 m (vehicle-m/day/km^2^)**	31.09	1.5 (0 - 201.0)

**Traffic Density within 300 m (vehicle-m/day/km^2^)**	28.42	1.6 (0 - 178.0)

**Traffic Density within 400 m (vehicle-m/day/km^2^)**	27.64	7.8 (0 - 156.5)

**DISTANCE TO AIRPORT-RELATED LOCATIONS**		

**Airport Terminal (m)**	1445.04	1454.0 (119 - 2724)

**Runway 16/34 (m)**	1051.36	1034.0 (68 - 2207)

**Runway 5/23 (m)**	704.46	649.0 (180 - 1774)

**Airport Centroid (m)**	1294.73	1245.0 (409 - 2483)

**Airport Fence (m)**	218.73	80.0 (0 - 1040)

**TRAFFIC-RELATED VARIABLES**		

**ADT-weighted road length within 100 m (vehicle-m/day)**	1063186.46	0 (0 - 7649264)

**ADT-weighted road length within 200 m (vehicle-m/day)**	3375258.66	0 (0 - 24119141)

**Length of Class 1 roads within 100 m (m)**	4.10	0 (0 - 299)

**Length of Class 1 roads within 200 m (m)**	17.63	0 (0 - 705)

**Length of Class 2 roads within 100 m (m)**	23.56	0 (0 - 267)

**Length of Class 2 roads within 200 m (m)**	82.47	0 (0 - 749)

**Length of Class 3 roads within 100 m (m)**	32.93	0 (0 - 493)

**Length of Class 3 roads within 200 m (m)**	114.51	0 (0 - 999)

**Length of Class 4 roads within 100 m (m)**	281.97	276.0 (0 - 800)

**Length of Class 4 roads within 200 m (m)**	990.32	990.0 (0 - 2394)

**Length of all roads within 100 m (m)**	342.55	361.0 (0 - 890)

**Length of all roads within 200 m (m)**	1204.94	1174.0 (0 - 2891)

Of the airport proxies, 'distance to the airport terminal' was most highly associated with measured NO_2 _in regressions including individual GIS covariates and session (Table 4). Statistical significance was also seen for distance to the primary airport runway (5/23), although with a positive coefficient (i.e., higher concentrations at greater distances). Neither 'distance to the airport centroid' nor 'distance to the airport fence line' was significantly associated with concentrations. Therefore, 'distance to the airport terminal' was retained as the primary airport proxy in subsequent model-building analyses.

**Table 4:** Associations between GIS variables and NO_2 _concentrations in models correcting for sampling session

Variable/Type	Estimate	Standard Error	t Value	P value
**DISTANCE TO NEAREST FUNCTION CLASS (RI DOT)**				

Distance to nearest Class 1 Road* (ppb/m)	-0.00236	0.000270	-8.75	< .0001

Distance to nearest Class 2 Road* (ppb/m)	-0.00296	0.000353	-8.38	< .0001

Distance to nearest Class 3 Road* (ppb/m)	-0.00477	0.000450	-10.61	< .0001

**Distance to nearest Class 4 Road (ppb/m)**	-0.00330	0.00178	-1.86	0.0639

**TRAFFIC DENSITY WITHIN VARIOUS RADII**				

Traffic Density within 100 m* (ppb-day-km^2^/(vehicle-m))	0.0155	0.00183	8.47	< .0001

Traffic Density within 200 m* (ppb-day-km^2^/(vehicle-m))	0.0235	0.00273	8.61	< .0001

Traffic Density within 300 m* (ppb-day-km^2^/(vehicle-m))	0.0327	0.00353	9.25	< .0001

Traffic Density within 400 m* (ppb-day-km^2^/(vehicle-m))	0.0427	0.00423	10.11	< .0001

**DISTANCE TO AIRPORT-RELATED LOCATIONS**				

Airport Terminal* (ppb/m)	- 0.00178	0.000289	- 6.15	< 0.0001

**Runway 16/34 (ppb/m)**	0.000331	0.000268	1.24	0.217

**Runway 5/23 (ppb/m)**	0.00138	0.000435	3.17	0.0016

**Airport Centroid (ppb/m)**	0.000344	0.000368	0.94	0.35

**Airport Fence (ppb/m)**	- 0.000379	0.000571	- 0.66	0.51

**TRAFFIC-RELATED VARIABLES**				

ADT-weighted road length within 100 m* (ppb-day/vehicle-m)	0.00000056	0.00000007	8.06	< 0.0001

ADT-weighted road length within 200 m* (ppb-day/vehicle-m)	0.00000024	0.00000003	8.64	< 0.0001

Length of Class 1 roads within 100 m* (ppb/m)	0.0325	0.00473	6.86	< 0.0001

Length of Class 1 roads within 200 m* (ppb/m)	0.0102	0.00169	6.05	< 0.0001

Length of Class 2 roads within 100 m*(ppb/m)	0.0169	0.00242	6.97	< 0.0001

Length of Class 2 roads within 200 m*(ppb/m)	0.00726	0.000934	7.78	< 0.0001

Length of Class 3 roads within 100 m*(ppb/m)	0.0159	0.00201	7.92	< 0.0001

Length of Class 3 roads within 200 m*(ppb/m)	0.00722	0.00081	8.95	< 0.0001

**Length of Class 4 roads within 100 m (ppb/m)**	- 0.000924	0.000716	- 1.29	0.20

**Length of Class 4 roads within 200 m (ppb/m)**	- 0.000411	0.000232	- 1.77	0.08

Length of all roads within 100 m*(ppb/m)	0.00297	0.000705	4.22	< 0.0001

Length of all roads within 200 m* (ppb/m)	0.000649	0.000220	2.95	0.0032

*Retained for multivariate model development, based on (session-corrected) univariate and bivariate analyses

Among variables representing distance to nearest road according to RI DOT classes, all coefficients were statistically significant (p < 0.001) except for Class 4 (minor) roads (p = 0.06). Traffic density variables were statistically significant across all buffer sizes (100 m through 400 m). Variables representing length of roadways within buffers surrounding the monitors also demonstrated statistical significance in most cases, with the exception of Class 4 roads. Table 4 only includes buffer sizes up to 200 m, as larger buffer sizes provided no additional explanatory value and were not considered in subsequent model-building. Variables created using buffer lengths of 100 m and 200 m were retained in our model development for roadways belonging to Classes 1, 2, and 3, as well as the length of all road segments (Table 4).

To construct our final multivariate models, we first included sampling session and the optimal measure of distance to airport-related locations ('distance to terminal'), and then tested the remaining variables to identify those with additional explanatory power. In general, the variables reflecting total roadway length were more robust and statistically significant in multivariate models than those representing distances to different road classes.

Our final multivariate model (Table 5) includes covariates representing the airport (as 'distance to terminal'), traffic density within 100 m of the sampling location, and attributes of Class 1, 2 and 3 roads (R^2 ^= 0.32). Class 1 and 2 roads are represented by the total road length within 100 and 200 m respectively, with the magnitudes of these coefficients corresponding well with the presumption that traffic volumes and source strengths are higher on Class 1 roads. The influence of Class 3 roads is represented by distance to the nearest member of this class.

**Table 5:** Results of general linear regression of predictors of NO_2 _near T.F. Green Airport (n = 644; R^2 ^= 0.32)

Variable	Estimate	Standard Error	t Value	P value	Partial R^2^
Intercept	12.64	0.51	24.99	< .0001	-

Session 1	2.18	0.35	6.32	< .0001	0.05
	
Session 2	1.89	0.31	6.14	< .0001	
	
Session 3	0.00	.	.	.	

Distance to terminal (m)	-0.00140	0.00026	-5.30	< .0001	0.10

Total traffic density within 100m	0.00830	0.0019	4.34	< .0001	0.05

Total length of Class 1 roads within 100 m (m)	0.0230	0.0044	5.21	< .0001	0.05

Total length of Class 2 roads within 200 m (m)	0.00381	0.00094	4.06	< .0001	0.03

Distance to nearest Class 3 road (m)	-0.00286	0.00047	-6.07	< .0001	0.04

We explored interactions between session and other covariates (reflecting differences in marginal contributions of near-field sources across seasons, potentially attributable to either meteorological differences or seasonal variations in source strength). In these models, we did not find significant interactions by season, with the exception of the effect estimates for Class 1 roadways and for the terminal effect. The Class 1 effect was strongest for Sessions 2 and 3, while the terminal effect was strongest during Session 1. Because of the lack of consistency in the relationship across covariates, and the fact that the effect estimates were not patterned in a manner consistent with likely seasonal patterns of emissions and meteorology, we did not consider this model to be interpretable, and retained the model in Table 5 as our final multivariate model.

**Figure 3:**
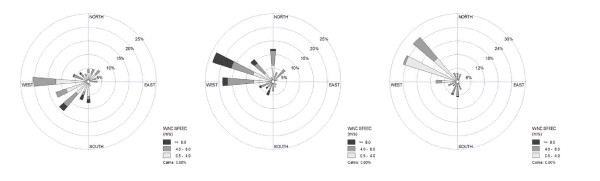
**Wind roses during three sampling campaigns: (a) October 2007, (b) March 2008 and (c) June 2008**.

We additionally evaluated the effect of session-specific meteorology on the associations with local traffic sources by repeating the model-building procedure using the wind-weighted class-specific roadway length variables (200 m buffers). As shown in Figure 3, winds were predominantly from the west, with a greater contribution from southwesterly winds in the fall and northwesterly winds in the spring and summer. The strong prevailing winds would seem to emphasize the importance of wind-weighted GIS covariates. However, the resulting models (not shown) did not have improved explanatory power relative to the model in Table 5 and did not increase the significance of the individual effect estimates. In fact, in models only including session and individual GIS variables (i.e., Table 4), the wind-weighted covariates demonstrated marginally weaker associations with NO_2 _concentrations for Class 2 and Class 3 roadways, with a modest improvement for Class 1 roadways that did not enhance multivariate model performance. We therefore did not include wind-weighting for any of our GIS-based covariates.

Within sensitivity analyses, we found general agreement with our final multivariate model across various model-building methodologies. An automated forward stepwise regression algorithm produced a model that included all variables presented in Table 5, with the addition of three covariates: traffic-weighted road length within 100 m and the total length of Class 3 variables within 200 m and 400 m. However, this model only provided modest improvement in explanatory power (R^2 ^= 0.34) and produced effect estimates that were counterintuitive for two of the variables (traffic-weighted road length and Class 3 roads within 400 m), likely influenced by the correlation between covariates. For example, the two additional Class 3 variables were highly negatively correlated with the distance to Class 3 roads variable (ρ = -0.62 and ρ = -0.67 for 200 m and 400 m, respectively), and the traffic-weighted road-length within 100 m was highly correlated with the total traffic density within 100 m (ρ = 0.96). Also, the inclusion of two Class 3 roadway length variables in the same model is problematic due to their strong positive correlation (ρ = 0.72). We also explored model-building using data from each individual session in separate models, to evaluate whether seasonal effects could result in not just different coefficients, but also different predictive covariates. There were some minor differences in the variables included in these models, but overall agreement in the explanatory categories represented (i.e., inclusion of terms for both traffic and airport proximity).

We also tested the sensitivity of our findings to our choice to provide dense sampling coverage along the airport's fence line, which contributed to non-uniform spatial coverage across the domain and could potentially bias our models. We separated our data set into "fence line" and "non-fence line" sampling points, and re-fit our final multivariate model. All individual effect estimates remained highly significant (p < 0.001) in both models, and the magnitudes of these estimates did not vary appreciably (differences < 30%) except for the covariate for distance from the terminal, which was approximately 50% smaller for the non-fence line dataset (which consisted of points at somewhat greater distances from the airport). These analyses suggest that our conclusions were not strongly influenced by our sampling design.

**Figure 4:**
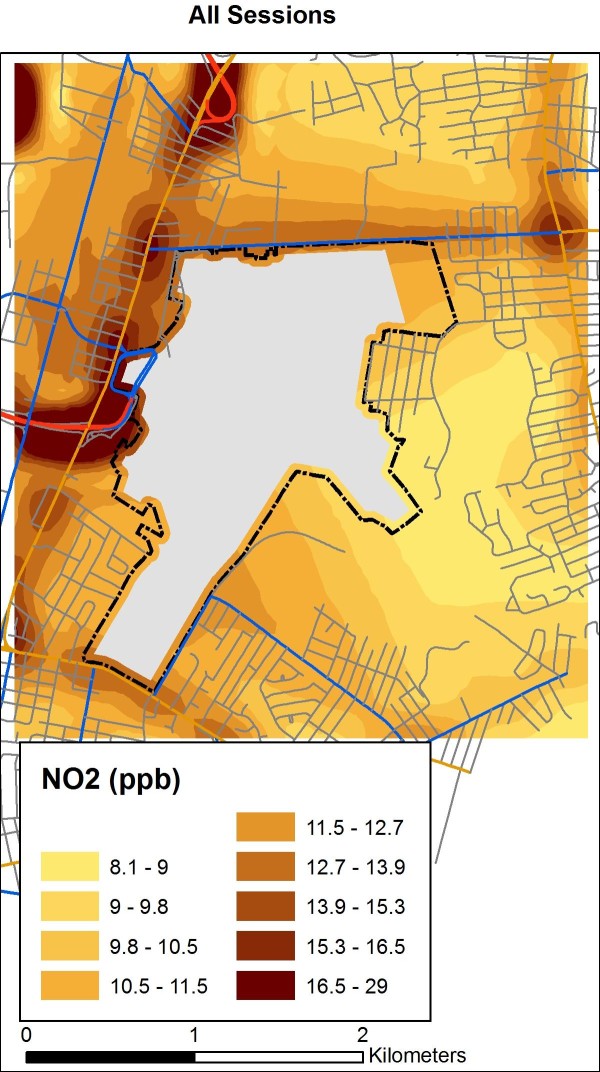
**Modeled NO_2 _concentrations at grid points spaced 200 m apart, averaged over three sessions**. (Surfaces were created using Radial Basis Functions. Cutpoints are quantiles of monitoring data from the October 2007 field campaign.)

Thus, our final multivariate model (Table 5) includes covariates for traffic density, distance to roadways, roadway length and distance to the airport terminal. Differences in units complicate comparisons of the marginal contribution from various sources. To facilitate interpretation of our model, we created a model-predicted surface using a 200 m grid over the sampling domain. As shown in Figure 4, the model captures key features seen in the smoothed surfaces of measured concentrations collected during each session.

**Figure 5:**
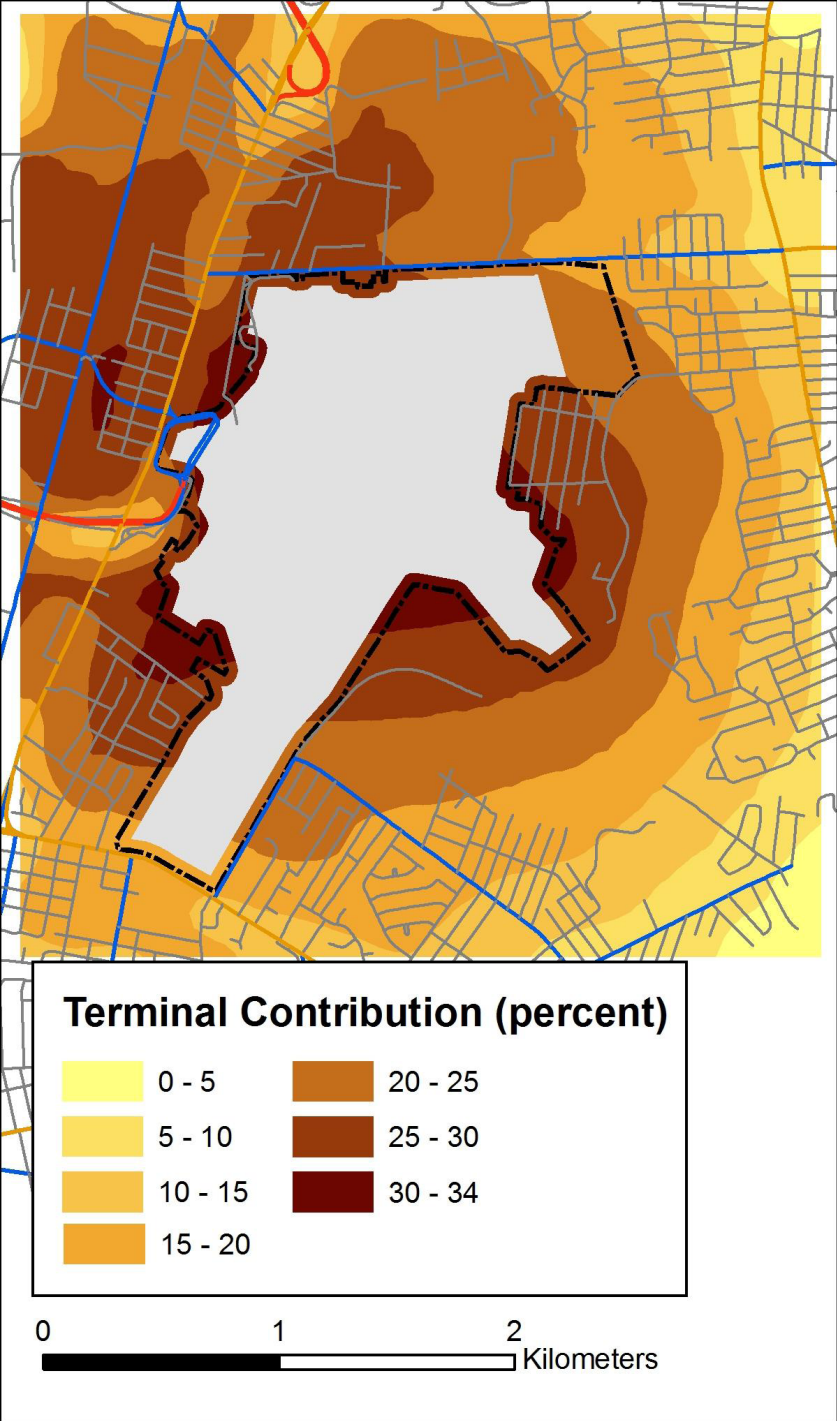
**Modeled contribution of the 'distance to airport terminal' parameter to average NO_2 _concentrations at grid points spaced 200 m apart, averaged over three sessions**. (Surfaces were created using Radial Basis Functions. Cutpoints are set to equal intervals.)

In addition, to approximate the marginal contribution of airport-related activities to local concentration patterns, we assumed that this was well represented by the 'distance to the airport terminal' effect estimate, recognizing that this covariate may proxy for multiple sources. To examine this effect, we assumed that the location in the domain farthest from the terminal had a null effect from the source represented by the 'distance to the airport terminal' variable, and we estimated the contributions from the terminal relative to this location. Using this approach, the sources represented by the 'terminal' covariate contributed up to 4.6 ppb (median over the domain = 2.4 ppb) to the total predicted NO_2_, averaged across the three sessions. In percentage terms, the 'terminal' covariate represented up to 34.4 percent (median over the domain = 21.4 percent) of the predicted NO_2 _contribution, with higher percentage contributions at locations near the airport but further from roadways. A smoothed surface of this relative contribution is shown in Figure 5.

## Discussion

Our study has shown that there are clear local variations in NO_2 _in the neighborhoods that surround an urban airport, which are spatially consistent across seasons. We have successfully used LUR modeling to explain a portion of this variability, which has several notable characteristics in our study area near T.F. Green Airport. Higher concentrations of NO_2 _are consistently seen near the terminal and entrance roads to the terminal. Local traffic has a strong influence on local variations in NO_2 _and independent effects are seen for all classes of these roads, except the smallest roads (Class 4) that predominate in residential areas.

While the 'airport' effect was evident in our multivariate analyses, the modeling does not have sufficient spatiotemporal resolution to allow us to separate the effects of ground operations from airport landing and takeoff (LTO) activity, or to separate either of these effects from near-terminal traffic that may be captured by the 'distance to terminal' covariate. Our observations are consistent with previous studies [14,15] which have provided evidence of the near-airport influence of LTO events on NOx as well as ultrafine particle and black carbon concentrations. The fact that the covariates for proximity to runways or the fence line were not significant in our analyses may be an indication that taxiing and ground support equipment are not significant contributors to the variability observed. The covariate for distance to the airport terminal may reflect a combination of parking lots, activities on nearby roadways, and aircraft and ground support activity proximate to the terminal, but our analyses could not separate out these contributions. The lack of sampling locations within the airport grounds may have also limited our ability to discern the individual contributions of ground operations and aircraft activity.

More generally, due to the high degree of correlation between many of our independent variables, it is difficult to separate the effects of airport activity from local traffic that is influenced by the presence of, as well as the activity within, the airport. To minimize spurious findings and enhance interpretability of our models, we focused on covariates representing distance to road classes rather than individual roadways. Had we followed the latter approach, the covariate for proximity to the airport terminal could be substituted with proximity to Interstate 95, with similar model performance but very different implications for source attribution. From the perspective of quantitative source apportionment, this is problematic. However, the relatively large distance between our sampling zone and Interstate 95 (approximately 1 mile) enhances the physical interpretability of our model, and more generally, our modeling highlights the multitude of connections between airport activity and local air pollution patterns. Clearly, the airport induces some local traffic, which then contributes to neighborhood-level exposures. Thus, the 'true' effect of the airport on local air pollution patterns is this traffic effect *plus *the pollutant emission patterns driven by ground operations and aircraft activity. This cumulative effect would be useful to understand for some analyses (e.g., characterizing the aggregate effects of airport expansion, evaluating the likelihood of future NAAQS violations), but targeted control strategies would clearly require more refined information available through atmospheric dispersion models or LUR models using more time-resolved source and concentration data.

We did not observe any improvements in explanatory power with the inclusion of GIS variables weighted by local wind patterns. This observation is likely associated with the spatial and temporal scales defined by our study. Within any urban environment, fine scale pollutant patterns may be difficult to describe in models built using pollutant and meteorological measurements averaged over one- to two-week sampling periods. Many factors influence this observation, including: local mixing and flow patterns around urban structures and mobile sources; heterogeneity in wind patterns across the study domain; and the short-term variations in source strength and wind direction and speed. More refined meteorological characterization could have been utilized, including consideration of diurnal patterns in source activity to upweight/downweight meteorological observations, but we lacked real-time traffic data concurrent with our sampling, and prevailing winds were generally quite consistent throughout the sampling day. More broadly, our goal in this study is to understand patterns of longer-term average exposure near the airport, which could be well characterized even in the absence of diurnal information.

An additional limitation of our LUR model is the fairly modest R^2 ^(0.32), which can be contrasted with R^2 ^on the order of 0.5-0.7 in many previous NO_2 _LUR models [5-7,9,11,12]. This is potentially attributable to multiple factors. First, most prior studies had a greater fraction of measurements near major roadways. In our study, fewer than 10% of the sample locations were within 200 m of a Class 1 roadway and fewer than 5% were within 100 m. While some studies [6] have shown comparable predictive power even when excluding measurements within 200 m of highways, these studies were in more urbanized and highly-trafficked settings than Warwick, and the airport itself is relatively small. More generally, although a majority of variability was unexplained by our multivariate regression model in Table 5, we were able to capture the key spatial features of NO_2 _concentrations in the neighborhoods of Warwick, as shown by Figures 2 and 4. The partial R^2 ^results in Table 5 also emphasize that the majority of explained variability is attributable to spatial covariates rather than the session dummy variable.

One of our study's strengths was the use of 'saturation' sampling, where a high sampling density is used across a relatively limited spatial domain. While relatively inexpensive for passive sampling of NO_2_, simultaneous deployment of 200-250 samplers is quite labor intensive, and it is valuable to understand whether less intensive efforts could have yielded comparable findings. To test the implications of smaller sample sizes, we repeated key analyses using randomly sampled subsets of our data representing one-quarter to one-half of our data. The effect estimate for Class 1 roadways was less stable at smaller sample sizes, likely due to the low density of Class 1 roadways across our study domain, implying that a relatively small proportion of our sampled sites contribute to the estimation of the effect of these roadways. (The low density of Class 1 variable may also explain its significant interaction with session.) This reinforces that optimal sampling would not randomly allocate samplers, and a preferred strategy would develop surfaces of proximity to major sources and oversample in grid cells near major roadways and/or containing large populations to capture adequate variability, as proposed and implemented elsewhere [16,17].

In contrast, effect estimates for the remaining variables in our final model were significant in all runs utilizing one-half of our data and were occasionally non-significant in models employing one-quarter of our data (minimum density tested). Individual effect estimates varied by 10-50% across random samples utilizing half of our data. When fewer sampling points are used, variability in these estimates increased, as expected. Effect estimates associated with the Class 1 variable were most likely to become non-significant in runs utilizing one-quarter of the data. These observations also highlight a factor related to the parameterization of our source variables. The choice to define narrow buffers for 'road length' variables is based on our knowledge of ground-level pollutant dispersion and the need to differentiate local influences across our domain (i.e., large buffers would yield convergent values in urban areas that are homogeneous at domain scales). However, these buffer-defined variables can limit the fraction of sampling points that contribute to parameter estimation where there is heterogeneity in source distribution at sub-domain scales (e.g., Class 1 roadways in our domain). Broadly, these analyses suggest that a reduced sample size with strategically deployed samplers would be adequate to characterize spatial variability in NO_2 _surrounding an airport, as found previously in relation to major roadways.

In general, any urban area with major roadways and numerous point/area sources presents some challenges when building and interpreting regression models designed to predict air pollution. We were cognizant of these challenges, and developed a model-building approach that could yield interpretable models subject to the constraints of available data. Our model-building focused on a number of summary traffic measures meant to capture both highway and surface traffic, and a small number of airport covariates that serve as proxies for the multitude of activities in and around the airport grounds. Of note, we did also attempt to construct covariates that would be physically interpretable, given that NO_2 _is known to display significant concentration gradients within a few hundred meters of the roadway [18]. For example, while Interstate 95 clearly has significant traffic and NO/NO_2 _emissions, it is located over a mile from the airport and would not likely contribute to a strong NO_2 _gradient across our domain (though it would elevate overall concentrations). Similarly, Providence (the closest major city) is approximately 8 miles away and would not contribute to small-scale gradients near the airport.

However, there remain challenges in interpreting key covariates, including the distance to terminal variable, which is arguably more amorphous than the traffic covariates. In our models, the 'terminal' effect may reflect many individual sources that cannot be discerned presently, such as: near-terminal traffic; activity in and around the parking lots and garages; emissions from equipment and vehicles associated with ground operations; and aircraft operations (taxiing, takeoff and landings). We have tried to avoid a narrow view of the meaning of the 'terminal' variable (i.e., that it represents only aircraft emissions) as well as the traffic variables (i.e., that they are separable from the airport's presence). More generally, it is also important to note that NO_2 _is only one component of the contributions from these sources; therefore, a full assessment of the health effects of local sources would require an understanding of similar patterns in several key pollutants, including particulate matter and hydrocarbons. Our modeling effort attempted to understand relative source contributions for potential design of control strategies, or more generally to sampling approaches in future studies. We also believe that this effort, linked with analyses of patterns of other pollutants, could inform future epidemiologic studies conducted in our study area.

## Conclusion

Our analysis, using LUR to model neighborhood-scale variations in nitrogen dioxide around an airport, has shown that variability is partially explained by seasonal differences and proximity to local combustion sources. Independent effects are seen for covariates representing traffic on larger roadways as well as a proxy for airport activity, 'distance to the airport terminal', although this variable may represent many factors beyond aircraft activity. In contrast, we found little evidence that activity on the airport grounds not proximate to the terminal contributed significantly to observed variability. Our results also suggest that more targeted sampling short of full saturation sampling may be adequate to capture dominant concentration patterns in proximity to an airport, especially given oversampling near particular sources and locations of interest.

## List of Abbreviations

EPA: Environmental Protection Agency; GIS: geographic information systems; GLM: general linear model; GPS: global positioning system; LTO: landing and take-off; LUR: land use regression; NAAQS: National Ambient Air Quality Standards; NO_2_: nitrogen dioxide; RI DOT: Rhode Island Department of Transportation; WHO: World Health Organization

## Competing interests

The authors declare that they have no competing interests.

## Authors' contributions

GA and JV supervised and assisted in field study operations. HH supervised preparation and analysis of passive samplers and assisted in field operations; SJM conducted all geocoding and the creation of GIS-based variables; GA conducted the statistical analysis and drafted the manuscript. JIL and JDS led the study design; JIL helped refine the analyses and revised the manuscript. All authors read and approved the final manuscript.

## References

[B1] Passchier W Public health impact of large airports Rev Environ Health 2000 15 83 96 10939086 10.1515/reveh.2000.15.1-2.83

[B2] Cohen BS Airport-related air pollution and noise J Occup Environ Hyg 2008 5 119 29 10.1080/15459620701815564 18097935

[B3] WHO Air quality guidelines - global update 2005 2006

[B4] U.S. EPA 40 CFR Parts 50 and 58: Primary National Ambient Air Quality Standards for Nitrogen Dioxide; Final Rule 2010 6474 6537

[B5] Brauer M Estimating long-term average particulate air pollution concentrations: application of traffic indicators and geographic information systems Epidemiology 2003 14 228 39 10.1097/00001648-200303000-00019 12606891

[B6] Gilbert NL The influence of highway traffic on ambient nitrogen dioxide concentrations beyond the immediate vicinity of highways Atmospheric Environment 2007 41 2670 2673 10.1016/j.atmosenv.2006.12.007

[B7] Ross Z Nitrogen dioxide prediction in Southern California using land use regression modeling: potential for environmental health analyses J Expo Sci Environ Epidemiol 2006 16 106 114 10.1038/sj.jea.7500442 16047040

[B8] Su JG An innovative land use regression model incorporating meteorology for exposure analysis Sci Total Environ 2008 390 520 529 10.1016/j.scitotenv.2007.10.032 18048083

[B9] Arain MA The use of wind fields in a land use regression model to predict air pollution concentrations for health exposure studies Atmospheric Environment 2007 41 3453 3464 10.1016/j.atmosenv.2006.11.063

[B10] Jerrett M Modeling the intraurban variability of ambient traffic pollution in Toronto, Canada J Toxicol Environ Health A 2007 70 200 212 10.1080/15287390600883018 17365582

[B11] Madsen C Modeling the intra-urban variability of outdoor traffic pollution in Oslo, Norway - A GA(2)LEN project Atmospheric Environment 2007 41 7500 7511 10.1016/j.atmosenv.2007.05.039

[B12] Rosenlund M Traffic-related air pollution in relation to incidence and prognosis of coronary heart disease Epidemiology 2008 19 121 128 10.1097/EDE.0b013e31815c1921 18091421

[B13] Palmes ED Personal sampler for nitrogen dioxide Am Ind Hyg Assoc J 1976 37 570 577 983946 10.1080/0002889768507522

[B14] Dodson RE An analysis of continuous black carbon concentrations in proximity to an airport and major roadways Atmospheric Environment 2009 43 3764 3773 10.1016/j.atmosenv.2009.04.014

[B15] Westerdahl D The Los Angeles International Airport as a source of ultrafine particles and other pollutants to nearby communities Atmospheric Environment 2008 42 3143 3155 10.1016/j.atmosenv.2007.09.006

[B16] Clougherty JE Land use regression modeling of intra-urban residential variability in multiple traffic-related air pollutants Environ Health 2008 7 17 10.1186/1476-069X-7-17 18485201 PMC2397396

[B17] Kanaroglou PS Establishing an air pollution monitoring network for intra-urban population exposure assessment: A location-allocation approach Atmospheric Environment 2005 39 2399 2409 10.1016/j.atmosenv.2004.06.049

[B18] Zhou Y Factors influencing the spatial extent of mobile source air pollution impacts: a meta-analysis BMC Public Health 2007 7 89 10.1186/1471-2458-7-89 17519039 PMC1890281

